# A Deletion in *FOXN1* Is Associated with a Syndrome Characterized by Congenital Hypotrichosis and Short Life Expectancy in Birman Cats

**DOI:** 10.1371/journal.pone.0120668

**Published:** 2015-03-17

**Authors:** Marie Abitbol, Philippe Bossé, Anne Thomas, Laurent Tiret

**Affiliations:** 1 U955 IMRB, INSERM, Équipe 10, Créteil, France; 2 BNMS—Génétique Médicale Comparée des Affections Neuromusculaires, École nationale vétérinaire d'Alfort, Maisons-Alfort, France; 3 Antagene, La Tour de Salvagny, France; University of Sydney, AUSTRALIA

## Abstract

An autosomal recessive syndrome characterized by congenital hypotrichosis and short life expectancy has been described in the Birman cat breed (*Felis silvestris catus*). We hypothesized that a *FOXN1* (*forkhead box N1*) loss-of-function allele, associated with the nude phenotype in humans, mice and rats, may account for the syndrome observed in Birman cats. To the best of our knowledge, spontaneous mutations in *FOXN1* have never been described in non-human, non-rodent mammalian species. We identified a recessive c.1030_1033delCTGT deletion in *FOXN1* in Birman cats. This 4-bp deletion was associated with the syndrome when present in two copies. Percentage of healthy carriers in our French panel of genotyped Birman cats was estimated to be 3.2%. The deletion led to a frameshift and a premature stop codon at position 547 in the protein. In silico, the truncated FOXN1 protein was predicted to lack the activation domain and critical parts of the forkhead DNA binding domain, both involved in the interaction between FOXN1 and its targets, a mandatory step to promote normal hair and thymic epithelial development. Our results enlarge the panel of recessive *FOXN1* loss-of-function alleles described in mammals. A DNA test is available; it will help owners avoid matings at risk and should prevent the dissemination of this morbid mutation in domestic felines.

## Introduction

Three breeds of domestic cats (*Felis silvestris catus*) are characterized by a non-syndromic congenital hypotrichosis. Sphynx cats are homozygous for an autosomal recessive hairless allele (*hr* or *Canadian hairless*) of the *KRT71* (*keratin 71*) gene [1]. Cats from the two related Donskoy and Peterbald breeds carry a semi-dominant hairless mutation, the molecular aetiology of which remains unknown [2]. Congenital hypotrichosis has also been regularly reported in other purebred and outbred cats since 1924 [3–9]. In the Birman breed, congenital hypotrichosis associated with reduced lifespan was described during the 1980’s, with hairless kittens born to two purebred Birman cats living in France and in the UK. None of the 13 reported hairless kittens survived beyond eight months; they died from respiratory or digestive infections, or were euthanized soon after birth for other unreported or unexplained medical reasons [3,10]. Necropsy and histopathological examination of nine hairless Birman kittens born to two normal parents living in Switzerland revealed an absence of the thymus and a lymphocyte depletion in the paracortical regions of the lymphoid tissue within spleen, Peyer’s patches and lymph nodes [11]. Analysis of Birman pedigrees suggested an autosomal recessive inheritance for this syndrome that associates congenital hypotrichosis and thymic aplasia [10,11]. About 50 years ago, a similar syndrome named the "nude" phenotype had been described in mice [12] and later reported in rats and humans [13,14]. It has also been referred to as the nude/SCID (inherited severe combined immunodeficiency) syndrome, which combines the hypotrichosis “nude” phenotype with an alymphoid cystic thymic dysgenesis causing T-cell immunodeficiency (SCID). Spontaneous loss-of-function mutations underlying nude phenotypes were identified in a member of the large *f* *orkhead b* *ox* gene family, encoding FOXN1 (forkhead box N1) [15,16]. From development to adulthood, FOX proteins are transcription factors involved in a variety of biochemical and cellular processes such as metabolism, aging or cancer. In the epidermis and the hair bulb, *FOXN1* is expressed at sites associated with the early stages of keratinocytes terminal differentiation, suggesting its implication in keratinocytes differentiation under proliferating conditions [17,18]. Within the thymus, *FOXN1* is expressed in epithelial cells and through the modulation of its target genes, promotes differentiation of immature epithelial cells into functional cortical and medullary thymic epithelial cells (TEC), which in turn are essential for development and selection of T-cells [15,19,20]. In the absence of FOXN1, epithelial differentiation is impaired, colonization of the thymic mesh by T cell progenitors from the bone marrow fails and finally T-cell and TEC formation aborts [19]. To the best of our knowledge, the nude syndrome has so far been restricted to humans [19,21–23], rats and mice [15]. Cases of hypotrichosis and thymic aplasia were previously reported in calves [24] and guinea pigs [25], however causal mutations have not been identified.

Here we report the identification of a *FOXN1* loss-of-function recessive allele in the Birman feline breed, associated with a syndrome combining hypotrichosis and short life expectancy.

## Results

### An autosomal recessive syndrome in Birman cats, combining hypotrichosis with short life expectancy

**Fig 1:**
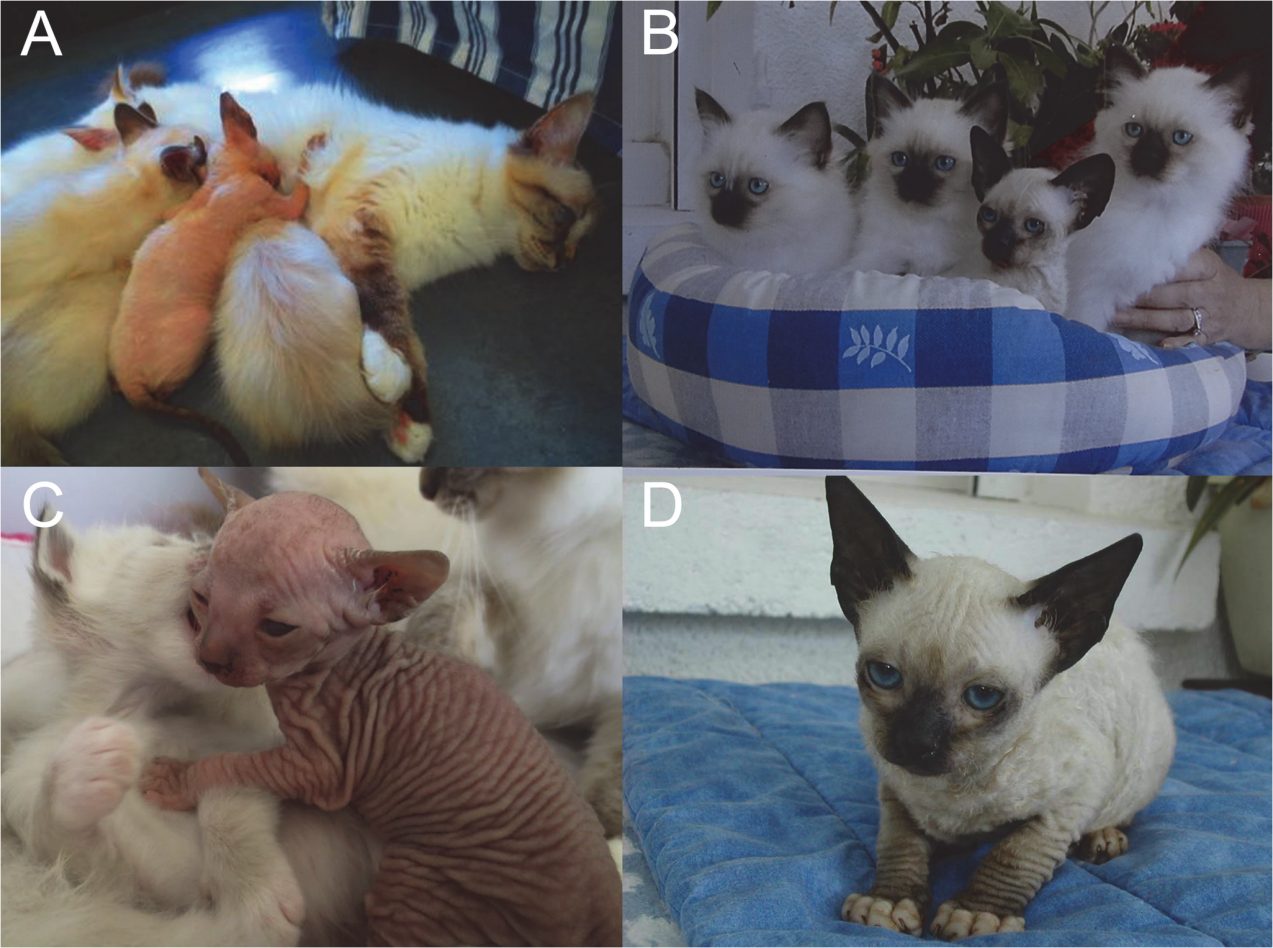
Hypotrichosis phenotype in Birman kittens. Hairless kittens among normal littermates, born to longhaired colourpoint mitted parents (A, B). A hairless 3-week-old kitten showing wrinkled skin (C). A 12-week-old hairless kitten displaying a sparse short fur with attenuated whiskers (D). Pictures A and C depict the same proband male, born in 2013. Pictures B and D depict a 12-week-old female kitten, born in 2004 and which is a proband relative.

In 2013, a male kitten (proband) was presented to the Genetic Consultation at the Alfort School of Veterinary Medicine, France. He was the only hairless kitten born in a litter of three males and two females (Fig. 1A) to two normal Birman parents. This male proband, from which genomic DNA had been extracted from previously swabbed cheek cells, died at home at four months of age, from severe diarrhoea. A pedigree snapshot revealed that his mother was related to a Birman male known to have produced in 2004 a litter of two males and two females including a hairless female (Fig. 1B). This female was euthanized at seven months of age because of skin infections, unfortunately no necropsy was performed and no biological sample was available.

**Fig 2:**
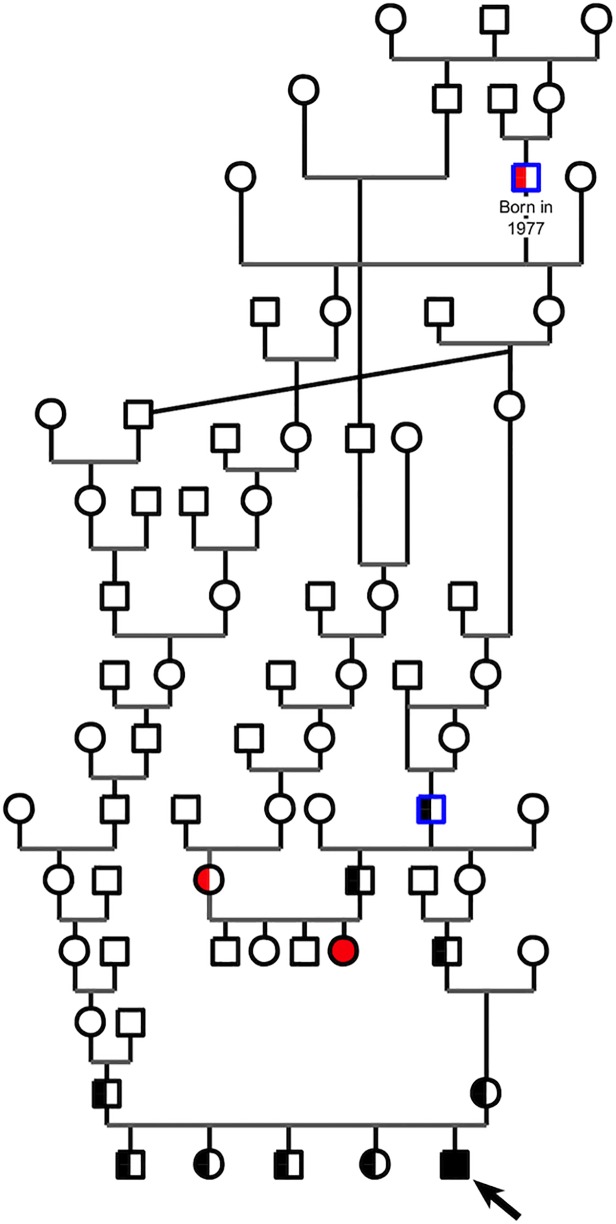
Pedigree-tree of a Birman cat family segregating hypotrichosis with reduced life span. Circles represent females, squares represent males. Affected kittens are depicted with fully filled symbols and the proband shown with an arrow. Healthy carriers are depicted with two-toned symbols. Red symbols represent cats with only phenotyping data (no DNA available); black symbols represent cats with phenotyping and genotyping data. For example, healthy cats with a *N/del* genotype are depicted with a two-toned black symbol. Common male ancestors of the two affected kittens are contoured in blue. This pedigree-tree suggests an autosomal recessive inheritance of the syndrome.

Both kittens were born hairless (e.g. in Fig. 1C) and developed sparse, shortened and fragile fur (Fig. 1D). Their skin was wrinkled and looked greasy. Besides, they were as active as their littermates and grew normally. A thorough pedigree analysis allowed to identify a common ancestor born in 1977, recognized by breeders to have produced several hairless kittens (Fig. 2). Pedigree data were consistent with an autosomal recessive inheritance pattern for this syndrome combining hypotrichosis with short life expectancy (Fig. 2).

### A candidate deletion in *FOXN1*

A first series of experiments consisted in screening the proband and his father for discriminating mutations in the forkhead box N1 (*FOXN1*) candidate gene, mutated in similar syndromes in mice, rats and humans. The *FOXN1* feline sequence was excerpted from Ensembl and used to design eight sets of primers (S1 Table) to cover the whole coding sequence. We successfully amplified the eight exons of the gene in the two cats, sequenced them and performed pair-wise base-to-base comparisons using Multalin. Seven single nucleotide polymorphisms (SNPs) and a 4-bp deletion were revealed between the two Birman cats and the Abyssinian feline reference sequence (Table 1). Five SNPs were synonymous mutations and two yielded non-synonymous mutations, predicted by PROVEAN to have no impact on FOXN1 function: p.(Ala136Asp); score −0.177 and p.(Val205Ala); score 0.343. The identified deletion (c.1030_1033delCTGT, exon 6) produced a frameshift leading to a premature stop codon at position 547 in the protein (Table 1). The proband carried two copies of this deletion and his father one copy, which was consistent with a recessive mode of inheritance of the aforementioned syndrome.

**Table 1:** Genomic variations in *FOXN1* identified between Birman exonic sequences and the Abyssinian coding reference sequence.

	c.407C>A exon 2	c.564G>A exon 2	c.614T>C exon 3	c.783T>C exon 4	c.1030_1033delCTGT exon 6	c.1356G>A exon 7	c.1665C>T exon 8	c.1776G>T exon 8
Ensembl feline sequence	C	G	T	T	N	G	C	G
Birman proband	*C/C*	*A/A*	*C/C*	*C/C*	*del/del*	*A/A*	*T/T*	*T/T*
Proband’s father	*C/A*	*A/A*	*C/C*	*C/C*	*N/del*	*A/A*	*T/T*	*T/T*
Consequence	p.(Ala136Asp)	p. (=)	p.(Val205Ala)	p. (=)	p.(Leu344Glyfs*203)	p. (=)	p. (=)	p. (=)
PROVEAN prediction	Neutral	-	Neutral	-	-	-	-	-

N: no deletion. *del*: CTGT deletion.

### The *FOXN1* deletion is associated with the syndrome

In a second series of experiments, a large cohort of 351 cats representing 16 breeds was genotyped for the mutated c.[1030_1033delCTGT] *FOXN1* allele. This cohort included the proband, 9 Birman cats from the proband family (Fig. 2), 126 unrelated Birman cats and 215 cats from 15 other breeds, among which cats from the Persian, Siamese and Oriental breeds that had been used to create and enlarge the genetic pool of the Birman breed in France (Table 2). The sire, dam, maternal grandfather and four normal littermates of the proband were all heterozygous for the mutated allele. In addition, DNA from the father and paternal grandfather of the affected female born in 2004 were available; we genotyped these two cats who also proved to be heterozygotes for the mutated allele (Fig. 2). We thus genotyped 215 cats from 15 non-Birman breeds and identified no cat carrying the deletion (Table 2). The perfect concordance between the recessively inherited syndrome in the analyzed Birman pedigree and the segregation within this pedigree of the specific c.1030_1033delCTGT deletion in *FOXN1* (Table 2) corroborated our hypothesis that the deletion was fully associated with the syndrome.

**Table 2:** Genotypes for the c.[1030_1033delCTGT] deletion in 16 breeds of cats.

Breed	*N/N*	*N/del*	*del/del*	Total	c.[1030_1033delCTGT] percentage
Control Birman cats	122	4	0	126	3.2%
Persian	29	0	0	29	0%
Siamese & Oriental SH and LH	16	0	0	16	0%
Ragdoll	14	0	0	14	0%
Total of 12 other breeds*	156	0	0	156	0%

* Chartreux (n = 21), Sphynx (n = 20), British SH and LH (n = 20), Norwegian Forest Cat (n = 20), Maine Coon (n = 15), Abyssinian and Somali (n = 15), Bengal (n = 13), Domestic SH and LH (n = 11), Devon Rex (n = 10), Siberian (n = 6), Russian and Nebelung (n = 4), Bombay (n = 1).

*N*: no deletion. *del*: CTGT deletion. SH: shorthair, LH: longhair

Finally, we assessed the percentage of cats carrying the c.[1030_1033delCTGT] *FOXN1* allele in a French panel of 126 Birman cats excluding first-degree relatives and found it to be 3.2% (Table 2).

### The *FOXN1* deleted allele is predicted to code for a truncated protein

**Fig 3:**
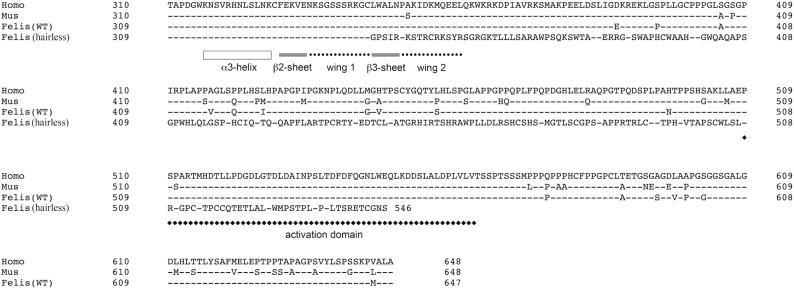
Wild type and mutant FOXN1 proteins. Alignment of partial amino acids sequences of FOXN1, translated from the wild type alleles reported in human (Homo, ENSP00000226247), mouse (Mus, ENSMUSP00000103929), cat [Felis (WT), ENSFCAP00000007665] and the c.[1030_1033delCTGT] mutated allele identified in hairless Birman cats (hairless). Amino acids encoded by exons 1 to 5 are omitted and alignment start with amino acid number 310 in human and mouse proteins, or amino acid number 309 in the feline protein. Human FOXN1 sequence was used as the reference sequence. Dashes represent identical amino acids compared to the reference sequence. FOXN1 structural features were depicted according to [26–29].

The c.1030_1033delCTGT deletion induces a frameshift leading to a premature stop codon at position 547 in the protein. To evaluate the putative functional impairment of this deletion on FOXN1, we aligned the truncated FOXN1 protein sequence with those of wild type FOXN1 proteins characterized in humans, mice and wild type cats. The global alignment (S1 Fig.) showed that feline and human wild type proteins displayed the highest homology level (92% identity), compared to 87% identity between the feline and mouse wild type proteins. In contrast, the predicted truncated FOXN1 protein lacked the last 101 amino acids and because of the frameshift, included a tail of 203 very divergent amino acids between position 344 and 546 (10% identity with the wild type protein; Fig. 3). According to the previously published consensus structure of forkhead transcription factors [26–29], the truncated, chimerical protein presumably produced in the affected Birman would lack the β3-sheet and second wing of the winged-helix DNA-binding domain (forkhead domain) and the complete activation domain (Fig. 3). Thus, the hypothesis supporting that FOXN1 function is impaired by the c.1030_1033delCTGT deletion was reinforced by these *in silico* structural data.

## Discussion

### The *FOXN1* deletion impairs the structural integrity of two functional domains

Although hypotrichosis and increased sensitivity to infections [3,10] or hypotrichosis with thymic aplasia [11] have been reported in the feline Birman breed for many years, the molecular aetiology of this syndrome has not yet been elucidated. Since a similar syndrome, called the nude syndrome, has been deciphered in mice, rats and humans and because the proband kitten died in its first months of age from a presumable intestinal infection, we opted for a candidate gene approach targeting the *FOXN1* gene which loss-of-function mutations had been shown to produce the nude phenotype in rodents and humans [15,20]. We identified eight polymorphisms in the exonic sequence of *FOXN1*, among which a c.1030_1033delCTGT deletion predicted to be a deleterious mutation; indeed the c.[1030_1033delCTGT] allele yields a truncated protein lacking the intact DNA binding and activation domains, both involved in FOXN1 function.

FOXN1 is a forkhead box protein belonging to an evolutionarily conserved family of transcription factors defined by a winged-helix DNA binding-domain, called the forkhead domain, composed of three α-helices, three β-strands and two loops (or wings). The third helix, known as the recognition helix, is involved in the interaction between the forkhead domain and the target DNA, while wings modulate DNA binding affinity and specificity [30]. Two nuclear localization sequences located in the first helix and in the second wing of the forkhead domain are responsible for the nuclear translocation of the protein. Notably, the C-terminal nuclear localization sequence in the second wing is highly conserved suggesting a common nuclear translocation mechanism shared by all forkhead transcription factors. Flanking regions of the winged-helix domain are poorly conserved among the forkhead box protein family and may contain effector domains [30]. More specifically, FOXN1 was found to contain an effector domain called the activation domain (AD) located in the C-terminal region of the protein. This conserved sequence is functionally critical for the transcriptional activation effect of FOXN1 [28,31]. Spontaneous loss-of-function mutations in the forkhead or the AD alone, or in the two of them in combination, were found to be responsible for a nude phenotype in rats and mice [16,28,31,32]. Importantly, the physical closeness between the two forkhead and AD is not only essential for the structural integrity of the protein; it has also been shown to promote target gene activation by FOXN1 [33].

The high similarity between feline and murine FOXN1 proteins (87% identity) was particularly striking in the forkhead domain (99% identity) and in the AD (98% identity). *In silico*, the c.1030_1033delCTGT deletion detected in two copies in the affected Birman kitten was predicted to produce a frameshift leading to a premature stop codon affecting the two forkhead domain and AD. A FOXN1 protein missing both the β3-sheet and the second wing of the forkhead domain likely cannot be translocated to the nucleus or bind DNA. Only 6/54 amino acids were conserved between the feline wild type and mutated AD. Additionally, the mutated AD was predicted to lack the last 15 amino acids (Fig. 3), suggesting that the mutated FOXN1 lacks a functional AD domain required to regulate the transcription of target genes. Altogether, these results strongly suggest that the mutated FOXN1 protein is not functional, and thus cannot promote skin and thymic epithelial development in the affected Birman cat.

### The *FOXN1* deletion is restricted to Birman cats

Genotyping of 215 cats from 15 non-Birman breeds for the *FOXN1* c.[1030_1033delCTGT] allele failed to identify carriers, showing a breed-restricted presence of the mutation in our cohort (Table 2). Despite no clear report describing the breed’s origin, Birman cats were initially bred in France at the beginning of the 20^th^ century and were first recognized in 1925 by the French *Cat Club de France* [34]. The Birman is a longhaired, colourpointed cat exhibiting a long silky coat with white gloves on each paw and blue eyes. The first Birman cats were obtained by crossing shorthaired colourpointed gloved Siamese cats with longhaired Persian cats [34]. Notably, none of the 16 Siamese/Oriental short/longhaired or of the 29 Persian/Exotic cats from our cohort carried the mutation. To the best of our knowledge, hairlessness has not been reported in the Persian breed. In contrast, hairlessness was previously reported in the Siamese breed, but the trait was not associated with reduced lifespan and hairless Siamese cats were able to breed. This suggests that *hairless* alleles segregating in the related Siamese and Birman breeds are different [7,10]. The two affected kittens and the nine healthy carriers from our family were related to a Birman tomcat born in 1977 and known by breeders to have produced several hairless kittens (Fig. 2). Therefore, we can hypothesize that the Birman *FOXN1* mutation arose after the creation of the breed in the 1920's and before 1977.

### The hairless Birman: a novel and potential large animal nude model?

Hypotrichosis was reported in various purebred and outbred cats but the syndrome associating hypotrichosis and sensitivity to infections, or thymic aplasia, has only been reported in the Birman breed ([11]; omia.angis.org.au). Although neither necropsy nor histopathological examinations could be performed in the affected kitten (proband), the breed, physical examination and premature death due to diarrhoea were all consistent with a diagnosis of hypotrichosis with thymic aplasia. Hence, we propose that hypotrichosis with short life expectancy, associated in the Birman breed with a recessive *FOXN1* loss-of-function mutation, would be assimilated to the nude phenotype. However, further characterisation of the histopathological phenotype of affected kittens is necessary to fully assess the identity of the syndrome associating hypotrichosis and reduced lifespan with the nude syndrome. Paucity of affected Birman kittens and their premature death have postponed this analysis.

Murine and rat spontaneous nude models have been extensively used to study epithelial development, immune function and cancer pathophysiology; they have also been used as preclinical models of therapies or xenograft transplantations [15,35–41]. To date, spontaneous as well as targeted engineered nude mice remain relevant models in immunology, haematology, cancer genesis and therapy [42]. However, limitations of their use in preclinical studies in cancer therapy have been identified because they often fail to predict response and effects of anticancer drugs in human patients [43]. As a consequence, there is a tremendous demand for more accurate animal models in this field, providing a more realistic set of physiological and outcome estimates. In the highly promising field of regenerative medicine, preclinical studies routinely assess potential effects of human autologous or allogenic stem cells transplanted into immunocompetent animal hosts. Such complex analyses are compromised by the interfering host-graft reaction, which alters therapy efficiency and complicates the interpretation of the results. Current approaches to bypass this pitfall include immunosuppression of the host animal and engineered immunodeficient animal lines or stem cell modification [44,45]. Accordingly, immunodeficient large animal models are expected to move regenerative medicine forward by combining permissiveness to cell transplantation and outcomes that can be better extrapolated to humans. This was recently highlighted by the characterization of spontaneous or RAG1/2 knockout immunodeficient swine stains [45,46].

The Birman syndrome described here, once fully confirmed at the histological or immunological level, would represent the first non-rodent mammalian nude model spontaneously mutated in *FOXN1*. Including this model in long-term preclinical studies would be relevant as it would be complementary to the pig which manipulation beyond the age of 6 months is hampered by its overweight. However, several issues such as the cost of husbandry, the specialized care and ethical issues related to the use of a companion animal in experimental procedures will have to be carefully considered.

## Conclusions

Our result enlarges the panel of recessive *FOXN1* loss-of-function alleles described in mammals and identified a likely non-rodent nude model.

Identification of the mutation underlying the hypotrichosis and short life expectancy syndrome in Birman cats will allow the prevention of matings at risk and avoid the birth of non-viable kittens. The DNA test resulting from this study is available to the community and will help breeders identify healthy carriers in their breeding stocks.

## Animals, Material and Methods

### Animals

Two hundred and sixty four cats from 16 breeds were included in the study. They were all sampled in France from February 2010 to March 2014 and included individuals from the following breeds: Birman (n = 49, including the proband and 9 cats from the proband’s family), Persian (n = 29), Chartreux (n = 21), Sphynx (n = 20), British shorthair and longhair (n = 20), Norwegian Forest (n = 20), Siamese and Oriental shorthair and longhair (n = 16), Maine Coon (n = 15), Abyssinian and Somali (n = 15), Ragdoll (n = 14), Bengal (n = 13), Domestic shorthair and longhair (n = 11), Devon Rex (n = 10), Siberian (n = 6), Russian and Nebelung (n = 4) and Bombay (n = 1). All cats were included at their owners’ request. Non-invasive buccal swabs were sent directly by owners or collected by a veterinarian. Birman cats were recruited specifically for the study. Non-Birman cats were recruited for genetic studies through a feline DNA banking initiative. Pedigrees were collected from the owners. Genealogical data were drawn using GenoPro (www.genopro.com).

DNA samples from 87 additional Birman cats, previously collected for DNA identification purposes using non-invasive buccal swabs, were used as controls.

### Ethics statement

All animals were client-owned cats on which no harmful invasive procedures were performed, so there was no animal experimentation according to the legal definition in Europe (Subject 5f of Article1, Chapter I of the Directive 2010/63/UE of the European Parliament and of the Council).

### DNA extraction

DNA was extracted from buccal swabs according to the manufacturers' protocols, using either a Maxwell 16 Instrument (Promega Corporation, Madison, USA), or the NucleoSpin 96 Tissue DNA Kit (Macherey-Nagel EURL, Hoerdt, France).

### FOXN1 sequencing and genotyping

Reference genomic sequences were excerpted from Ensembl [www.ensembl.org; feline *FOXN1* gene, (ENSFCAG00000008268)]. PCR and sequencing primers were designed using Primer3 [47]. Exonic genomic sequences were amplified and sequenced using primers from S1 Table. Exons were individually amplified for each cat from 100 ng of their genomic DNA according to the manufacturers’ protocol, with Q-Bio Taq DNA Polymerase (Qbiogen MP Biomedicals Inc., Carlsbad, CA). Four hundred ng of each PCR amplicon were sent to GATC Biotech (GATC Biotech AG, Konstanz, Germany); there, they were purified and Sanger sequenced in both forward and reverse directions. Electropherograms were manually inspected with Chromas Lite (Technelysium Pty Ltd, South Brisbane, Australia). Multiple alignments were performed using Multalin ([48]; http://multalin.toulouse.inra.fr/; identity matrix).

### Protein sequence comparisons and structural prediction

Cat, human and mouse FOXN1 sequences were collected from Ensembl [www.ensembl.org; cat: (ENSFCAP00000007665), human: (ENSP00000226247) and mouse: (ENSMUSP00000103929)]. Multiple alignments were performed using Multalin ([48]; http://multalin.toulouse.inra.fr/; BLOSUM-62 and identity matrix). The predicted impact of missense mutations was assessed using PROVEAN ([49]; provean.jcvi.org/seq_submit.php). FOXN1 structural features were depicted according to [26–29].

### Accession numbers

Genomic coding sequences of *FOXN1* exon 6 from longhaired (wild type) and hairless (mutated) Birman cats (*Felis silvestris catus*) were submitted to GeneBank. Accession numbers are (GenBank: KJ849281) for the wild type allele and (GenBank: KJ849282) for the c.[1030_1033delCTGT] mutated allele.

## Supporting Information

S1 Fig Alignment of the predicted mutant FOXN1 protein with full-length wild type orthologs. Alignment of amino acids sequences of FOXN1, translated from the wild type alleles reported in human (Homo, ENSP00000226247), cat [Felis (WT), ENSFCAP00000007665], mouse (Mus, ENSMUSP00000103929), and the c.[1030_1033delCTGT] mutated allele identified in the hairless Birman kitten (hairless). Human FOXN1 sequence was used as the reference sequence. Identical amino acids in the four sequences are depicted in red. Points represent identical amino acids compared to the reference sequence. Dashes represent deletions.
(TIFF)

S1 Table PCR and sequencing primers. (DOCX)
