# Supplementary figures and images for: A Deletion in FOXN1 Is Associated with a Syndrome Characterized by Congenital Hypotrichosis and Short Life Expectancy in Birman Cats
Source: PLoS One. 2015 Mar 17;10(3):e0120668. doi: 10.1371/journal.pone.0120668 (PMC4363148; doi:10.1371/journal.pone.0120668)

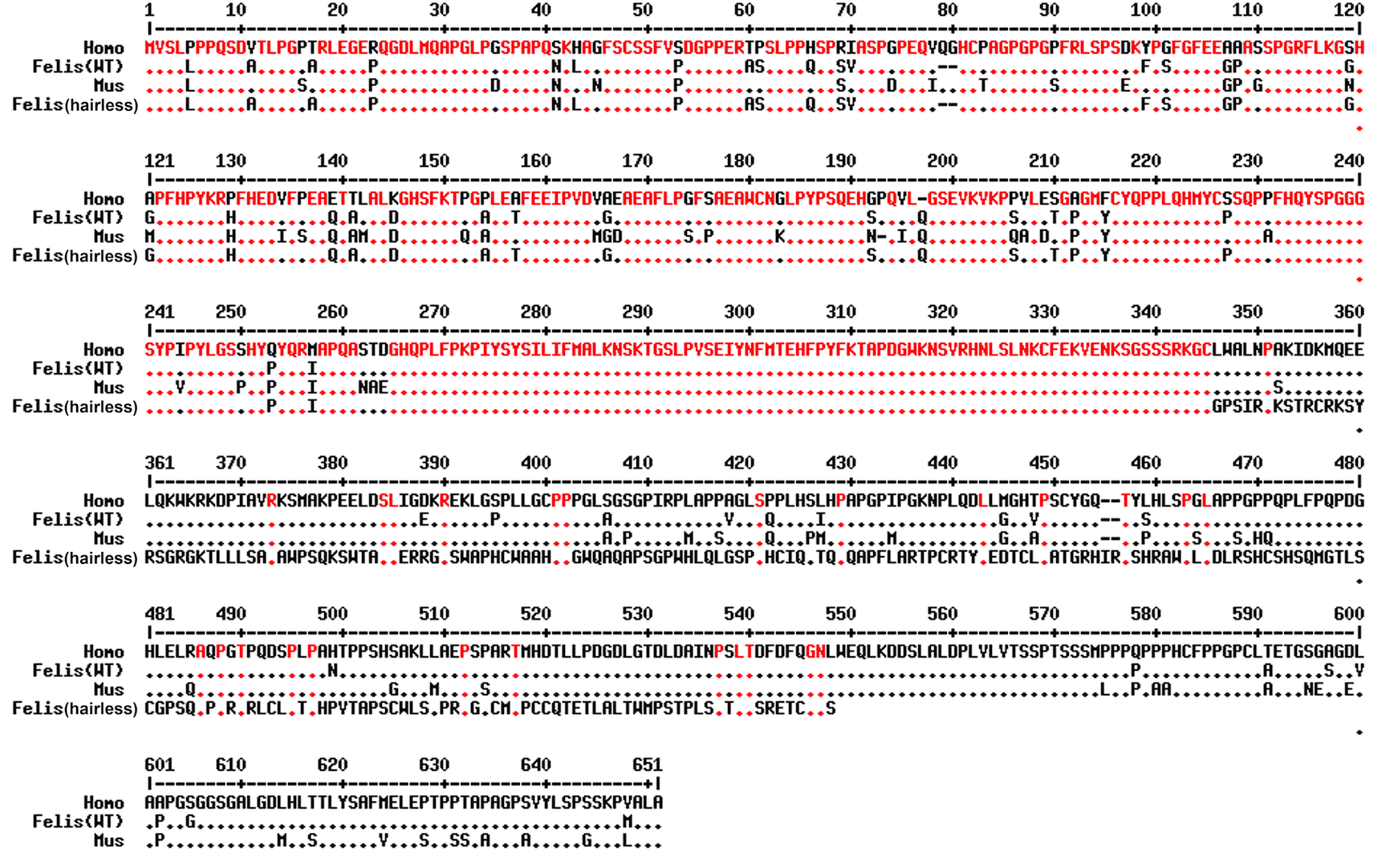

Supplement: S1 Fig — Alignment of amino acids sequences of FOXN1, translated from the wild type alleles reported in human (Homo, ENSP00000226247), cat [Felis (WT), ENSFCAP00000007665], mouse (Mus, ENSMUSP00000103929), and the c.[1030_1033delCTGT] mutated allele identified in the hairless Birman kitten (hairless). Human FOXN1 sequence was used as the reference sequence. Identical amino acids in the four sequences are depicted in red. Points represent identical amino acids compared to the reference sequence. Dashes represent deletions. (TIFF) [file pone.0120668.s001.tiff]
