# Supplementary material for: A Deletion in FOXN1 Is Associated with a Syndrome Characterized by Congenital Hypotrichosis and Short Life Expectancy in Birman Cats
Source: PLoS One. 2015 Mar 17;10(3):e0120668. doi: 10.1371/journal.pone.0120668 (PMC4363148; doi:10.1371/journal.pone.0120668)
Supplement: S1 Table — (DOCX) [file pone.0120668.s002.docx]

**Table S1. PCR and sequencing primers**

| **Exon** | **Forward primer** | **Forward primer sequence** | **Tm** | **Reverse primer** | **Reverse primer sequence** | **Tm** | **PCR product** |
| --- | --- | --- | --- | --- | --- | --- | --- |
| *FOXN1*exon1 | *FOXN1*exon1F | GGTCTGGTGGGAGTCAGG | 60.5°C | *FOXN1*exon1R | TGGAAGGGAGAACCAGAGG | 58.8°C | 386 bp |
| *FOXN1*exon2 | *FOXN1*exon2F | GGAGCTCAGCCACAGACC | 60.5°C | *FOXN1*exon2R | AAGGGAAGGCGGAGTTTG | 56°C | 743 bp |
| *FOXN1*exon3 | *FOXN1*exon3F | TTTGGGGGAGGCAGAGTAG | 58.8°C | *FOXN1*exon3R | TGTGCACACGCACAACAC | 56°C | 563 bp |
| *FOXN1*exon4 | *FOXN1*exon4F | TGGAGCCCATGACCAGAG | 58.2°C | *FOXN1*exon4R | GAAACCCACGGGGTATGAC | 58.8°C | 396 bp |
| *FOXN1*exon5 | *FOXN1*exon5F | TTCCTGGCTTCAGCGAAC | 56°C | *FOXN1*exon5R | ATGGAGACCAGGGGAAGC | 58.2°C | 429 bp |
| *FOXN1*exon6 | *FOXN1*exon6F | ACGGGTGGCAGTTCTGTG | 58.2°C | *FOXN1*exon6R | CTCAGGCCCTCCCCTTAC | 60.5°C | 446 bp |
| *FOXN1*exon7 | *FOXN1*exon7F | GTAAGGGGAGGGCCTGAG | 60.5°C | *FOXN1*exon7R | TGCAGCCTGGAGACTTGG | 58.2°C | 673 bp |
| *FOXN1*exon8 | *FOXN1*exon8F | CTCCCCAGCCAAGGTTAC | 58.2°C | *FOXN1*exon8R | AAAGGCTTCTGGGTGTGG | 56°C | 457 bp |
